# Genome sequence of *Shigella flexneri* strain SP1, a diarrheal isolate that encodes an extended-spectrum β-lactamase (ESBL)

**DOI:** 10.1186/s12941-017-0212-2

**Published:** 2017-05-12

**Authors:** Ping Shen, Jianzhong Fan, Lihua Guo, Jiahua Li, Ang Li, Jing Zhang, Chaoqun Ying, Jinru Ji, Hao Xu, Beiwen Zheng, Yonghong Xiao

**Affiliations:** 1 Collaborative Innovation Center for Diagnosis and Treatment of Infectious Diseases, State Key Laboratory for Diagnosis and Treatment of Infectious Diseases, The First Affiliated Hospital, School of Medicine, Zhejiang University, Hangzhou, 310003 China; 2 Department of Clinical Laboratory, Hangzhou First People’s Hospital, Hangzhou, 310006 China; 3 Department of Hospital Infection Control, Zhucheng People’s Hospital, Zhucheng, 252300 China

**Keywords:** *Shigella flexneri*, Extended-spectrum β-lactamase, IncFII2, Comparative genomic analysis

## Abstract

**Background:**

Shigellosis is the most common cause of gastrointestinal infections in developing countries. In China, the species most frequently responsible for shigellosis is *Shigella flexneri*. *S. flexneri* remains largely unexplored from a genomic standpoint and is still described using a vocabulary based on biochemical and serological properties. Moreover, increasing numbers of ESBL-producing *Shigella* strains have been isolated from clinical samples. Despite this, only a few cases of ESBL-producing *Shigella* have been described in China. Therefore, a better understanding of ESBL-producing *Shigella* from a genomic standpoint is required. In this study, a *S. flexneri* type 1a isolate SP1 harboring *bl*a_CTX-M-14_, which was recovered from the patient with diarrhea, was subjected to whole genome sequencing.

**Results:**

The draft genome assembly of *S. flexneri* strain SP1 consisted of 4,592,345 bp with a G+C content of 50.46%. RAST analysis revealed the genome contained 4798 coding sequences (CDSs) and 100 RNA-encoding genes. We detected one incomplete prophage and six candidate CRISPR loci in the genome. In vitro antimicrobial susceptibility testing demonstrated that strain SP1 is resistant to ampicillin, amoxicillin/clavulanic acid, cefazolin, ceftriaxone and trimethoprim. In silico analysis detected genes mediating resistance to aminoglycosides, β-lactams, phenicol, tetracycline, sulphonamides, and trimethoprim. The *bla*_CTX-M-14_ gene was located on an IncFII2 plasmid. A series of virulence factors were identified in the genome.

**Conclusions:**

In this study, we report the whole genome sequence of a *bl*a_CTX-M-14_-encoding *S. flexneri* strain SP1. Dozens of resistance determinants were detected in the genome and may be responsible for the multidrug-resistance of this strain, although further confirmation studies are warranted. Numerous virulence factors identified in the strain suggest that isolate SP1 is potential pathogenic. The availability of the genome sequence and comparative analysis with other *S. flexneri* strains provides the basis to further address the evolution of drug resistance mechanisms and pathogenicity in *S. flexneri*.

**Electronic supplementary material:**

The online version of this article (doi:10.1186/s12941-017-0212-2) contains supplementary material, which is available to authorized users.

## Background

*Shigella* species are a major causative cause of gastrointestinal infections throughout the world, especially in developing countries [1]. Globally, there are approximately 164.7 million cases per annum, of which 1.1 million people are estimated to die from *Shigella* infections [2]. Based on biochemical and serological properties, the genus *Shigella* comprises four serogroups: *Shigella dysenteriae*, *Shigella flexneri*, *Shigella boydii* and *Shigella sonnei* [2]. *S. flexneri* is endemic in many developing countries and causes more deaths than any other *Shigella* serotypes [3]. In China, shigellosis is the most common gastrointestinal infections [4] and most frequently isolated species responsible for shigellosis in mainland China is *S. flexneri* [5].

Antibiotic treatment is usually recommended for shigellosis as it reduces the duration and severity of symptoms, reduces the excretion of organisms and prevents potentially lethal complications [6]. However, the emergence of extended-spectrum β-lactamase (ESBL)-producing *S. flexneri* is a major public health problem in China, as these strains are associated with critical infections [7]. Most of the CTX-M, SHV, and TEM-type ESBLs genes are located on conjugative plasmids [4]. Moreover, co-existence with other antimicrobial resistance genes is frequently observed in ESBL-producers [8], which makes the choice of effective treatment extremely limited. Increasing instances of ESBL-producing *Shigella* strains isolated from Asia have been reported [1, 2]. So far, only a few cases of ESBL-producing *Shigella* have been described in China [4]. Therefore, a better understanding of ESBL-producing *Shigella* using a genomics approach is required.

The first genome sequence of *S. flexneri* was reported by Jin et al. in 2002 [9]. So far, more than 145 *S. flexneri* strains have been sequenced and analyzed [10]. To extend our understanding of the resistance mechanisms and pathogenesis of ESBL-producing *S. flexneri*, we performed sequencing and genomic analysis of the ESBL-harbouring *S. flexneri* SP1. Comparative genomics analysis of *S. flexneri* SP1 with other *S. flexneri* genomes may improve our understanding of the antibiotic resistance and virulence factors present in *Shigella*.

## Methods

### Strain information

*Shigella flexneri* 1a isolate SP1 was isolated from the stool sample of a 76-year-old female diarrhea patient. Standard biochemical tests were performed using the Vitek II system (BioMerieux, France) and species-specific 16S rRNA sequencing was used to confirm the identity of isolate SP1. Serotyping was performed with specific antiserum (Denka-Seiken). Genomic DNA of SP1 was extracted from a single colony of the pure bacterial culture. Possible contamination with other DNA and misassemblies were assessed by performing a BLAST search against the non-redundant database as described previously [11]. The whole genome of SP1 is in the expected size range for a *Shigella* genome and the coverage of the reads was consistent throughout the genome. The assembled draft genome sequence of SP1 was further verified by comparative analysis with the published complete genome sequences of *S. flexneri* strains.

### Antimicrobial susceptibility testing

Susceptibility testing for ampicillin, amoxicillin/clavulanic acid, amikacin, aztreonam, piperacillin/tazobactam, cefazolin, cefoxitin, ciprofloxacin, ceftriaxone, cefepime, ertapenem, imipenem, gentamycin, tobramycin, levofloxacin, tigecycline, nitrofurantoin, and trimethoprim were performed by the microbroth dilution method and interpreted according to the CLSI guidelines [12]. *Escherichia coli* strain ATCC 25922 was used as the control strain for susceptibility studies. Late log phase cells were harvested, and genomic DNA was extracted from the pure culture using the DNeasy Blood & Tissue kit (Qiagen, Germany) according to the manufacturer’s instructions. For the purpose of bacterial identification, we amplified the 16S rRNA gene with a 16S rRNA universal primer set and the PCR product was sequenced. Ethical approval was granted by the Ethics Committee of the First Affiliated Hospital of Zhejiang University.

### Genome sequencing and assembly

The extracted DNA was visualized by agarose gel electrophoresis and quantitated by Qubit 2.0. Whole-genome sequencing was performed on the Illumina HiSeq 4000-PE150 platform. DNA was tailed, ligated to paired-end adaptors and PCR amplified with a 500 bp insert size and a mate-pair libraries with an insert size of 5 kb were used for library construction at the Beijing Novogene Bioinformatics Technology Co., Ltd. Illumina PCR adapter reads and low quality reads from the paired-end and mate-pair library were filtered by the quality control step using Novogene pipeline. All high quality paired reads were assembled using Velvet 1.2.10 [13] into a number of scaffolds. The filtered reads were then passed handled by the next step of the gap-closing.

### Genome annotation

Genome annotation included the prediction of coding genes, transfer RNAs, ribosomal RNA, prophage, and clustered regularly interspaced short palindromic repeat sequences (CRISPR). Open reading frames (ORFs) were identified and classified using the Rapid Annotation using Subsystem Technology (RAST) server [14]. Protein classification into functional groups was performed using the Clusters of Orthologous Groups of proteins (COGs) [15]. Transfer RNAs and ribosomal RNA genes rRNAs were detected by tRNAscan-SE [16] and RNAmmer 1.2 software [17], respectively. PHASTER [18] was used to identify prophage and putative phage-like elements and CRISPRFinder [19] was used to identify CRISPR sites. The plasmid replicon was predicted by the PlasmidFinder Tool [20]. ISfinder [21] was employed to search for IS sequences in the genome, with an e-value of 1E−3. plasmidSPAdes was used to produce plasmid sequences from the WGS data [22].

### Antibiotic resistance genes prediction and virulence factors analysis

Antibiotic resistance genes were annotated using the comprehensive antibiotic resistance database (CARD) [23] and Resfinder [24] with default parameters. We further verified all putative antibiotic resistance genes (ARGs) through a BLAST search with cut-off e-value of 1E−0.5. Virulence factors were predicted by using BLAST to search against the VFDB database [25] with an e-value threshold of 1E−5 and also with VirulenceFinder 1.5 [26].

### Plasmid characterization

PlasmidFinder 1.3 was used for identify the incompatibility group of the plasmid present in *S. flexneri* SP1 [20]. The plasmid sequence of *bla*_CTX-M-14_-harboring plasmid from isolate SP1 (named pSP1) was assembled with plasmidSPAdes [22]. Assignment of the plasmid to an incompatibility (Inc) group was performed by multiplex PCR. PCRs were performed as described previously [27].

### Phylogenetic analysis and comparative genomic analysis

Comparative genomic analysis was performed by orthology identification method as previously described [11, 28]. Genome sequences of the following representative *S. flexneri* strains were downloaded from the NCBI genome database: *S. flexneri* 2a strain 981 (CP012137), *S. flexneri* S7737 (AMJY00000000), *S. flexneri* 5a strain M90T (CM001474), *S. flexneri* CDC 796.83 (AERO00000000), *S. flexneri* 4343.70 (AFHC00000000), *S. flexneri* NCTC1 (LM651928), *S. flexneri* 2a strain 301 (AE005674), *S. flexneri* G1663 (CP007037), *S. flexneri* Shi06HN006 (CP004057), *S. flexneri* 2003036 (CP004056), *S. flexneri* str 4S BJ10610 (JMRK00000000), *S. flexneri* 4c strain 1205 (CP012140), *S. flexneri* 2002017 (CP001383) and *S. flexneri* 1a strain 0228 (CP012735). Phylogenetic reconstruction and analysis was performed wih the phangorn package, written in the R language [29]. VennDiagram [30] was used to generate the Venn plots of *S. flexneri* SP1, *S. flexneri* str 4S BJ10610, *S. flexneri* 4c strain 1205 (CP012140), *S. flexneri* 2002017, and *S. flexneri* 1a strain 0228.

## Results and discussion

### General features

**Fig. 1:**
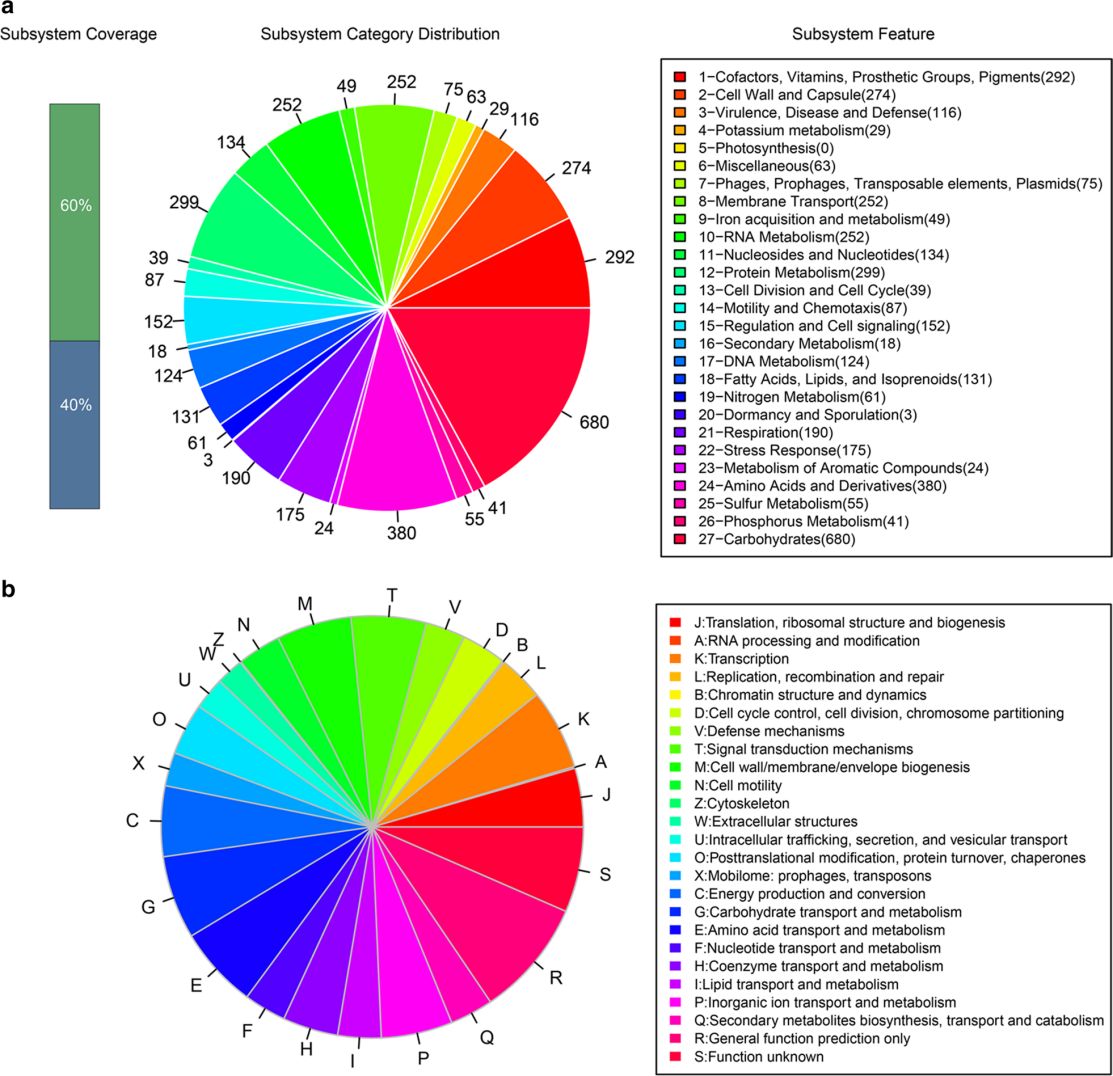
Analysis of annotated genes in *S. flexneri* strain SP1 based on the SEED and COG databases. **a** The *green bar* represents the percentage of proteins that were annotated by RAST server, while the *blue bar* indicated the proteins not annotated. The* pie chart* demonstrates the abundance of each subsystem category and the count of each subsystem feature is shown. **b** Distribution of COGs. *Each bar* indicates the number of annotated genes based on the COG database

We performed whole genome sequencing using the Illumina HiSeq 4000 system with 2 × 150 bp paired-end reads. After quality control, we assembled the 1095 M bp filtered reads into contigs. The assembled genome of *S. flexneri* SP1 revealed a genome size of 4,592,345 bp with a G+C content of 50.46%. The largest contig consisted of 137,097 bp and the length of N50 contig was 33,394 bp. These scaffolds contain 4798 coding sequences (CDSs), and 100 RNA-encoding genes. The properties and the statistics of the genome are summarized in Additional file 1: Table S1. The resulting genomic size of strain SP1 was similar to previous studies within the range of 4.1–4.8 M bp [31, 32]. Similarly, CDSs numbers were close to the previous publication [31]. Gene functions were predicted using RAST and COG analysis. RAST server based annotation of the whole genome describes the distribution of subsystems in strain SP1 (Fig. 1a). Proteins responsible for carbohydrates (693 ORFs), amino acids and derivatives (384 ORFs), and cofactors, vitamins, prosthetic groups, pigments (292 ORFs) were abundant among the subsystem categories. The distribution of COGs is illustrated in Fig. 1b. The most abundant COG categories were R (general function prediction only), S (function unknown), E (amino acid transport and metabolism), G (carbohydrate transport and metabolism) and K (transcription). Furthermore, one incomplete prophage region was identified in the genome of SP1. It is a *Salmonella* ST64B-like phage (Acc-No. NC_004313) of 14.1 kb in length and a G+C content of 50.81%. Additionally, six questionable CRISPR loci were detected by CRISPERfinder.

### Antimicrobial susceptibility profiles and antibiotic resistance genes

The in vitro antimicrobial susceptibility testing demonstrated that the strain SP1 was resistant to ampicillin, amoxicillin/clavulanic acid, cefazolin, ceftriaxone and trimethoprim, but susceptible to piperacillin/tazobactam, cefoxitin, cefepime, aztreonam, imipenem, amikacin, gentamicin, tobramycin, ciprofloxacin, levofloxacin, tigecycline and nitrofurantoin (Additional file 2: Table S2). We then screened the antibiotic resistance genes (ARGs) in the genome to further explore the genetic basis of multidrug resistance in this strain (Additional file 3: Table S3). In silico analysis revealed the presence of some putative ARGs for different drug classes. We detected genes mediating resistance to aminoglycosides (*aadA24*, *strA* and *strB*), β-lactams (*bla*_CTX-M-14_ and *bla*_OXA-1_), phenicol (*catA1*), tetracycline (*tetD*), sulphonamides (*sul2*), and trimethoprim (*dfrA1*). CTX-M-14 was the most frequent ESBL variant detected in *Shigella* isolates in China, followed by CTX-M-15 [4, 33]. Hitherto, only a few studies have reported the presence of ESBLs in *S. flexneri* [34]. Interestingly, all of the *bla*_CTX-M-14-_harbouring *S. flexneri* strains were isolated from China [4, 7, 34, 35]. A previous study reported a high prevalence of extended-spectrum cephalosporin resistance *Shigella* mediated mainly by *bla*_CTX-M_ (mainly *bla*_CTX-M-14_, 14.1%) in Hangzhou City, Zhejiang Province, China [35]. Our study further indicates the existence of *bla*_CTX-M-14-_harbouring *S. flexneri* clone may responsible for these *Shigella* infections in this area. In addition, the insertion sequence (IS) elements were frequently detected in upstream of *bla*_CTX-M_ genes [36]. ISfinder was thus employed to scan the *bl*a_CTX-M-14_ flanking sequences in a range of 6-kb for IS sequences and junction associated proteins. IS*Ecp1* and IS903B were found upstream of *bl*a_CTX-M-14_. IS*Ecp1* belongs to the IS1380 family, which may enhance the expression of *bla*_CTX-M-14/-18_, *bla*_CTX-M-17_ and *bla*_CTX-M-19_ β-lactamase genes [37].

### Genetic context of *bla*_CTX-M-14_ gene

**Fig. 2:**
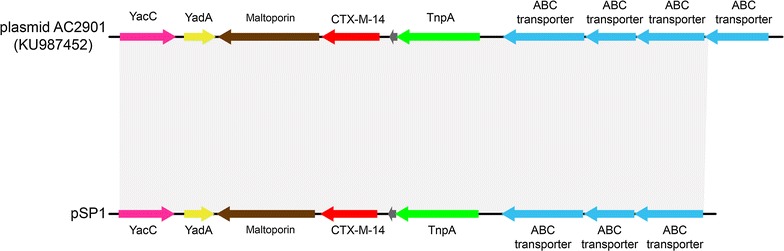
Genetic organization of scaffolds (portions of genome sequences reconstructed from the whole-genome sequence) containing *bla*_CTX-M-14_ harbored by plasmid pSP1 and structural comparison with plasmid pAC2901. *Arrows* indicate positions and direction of transcription of genes. Regions with >99% homology are shown in* gray*. Information in parentheses after isolates represents the GenBank accession number

PlasmidFinder and plasmidSPAdes were used to detect the potential plasmids in the whole genome sequence. *In silico* analysis revealed that *bla*_CTX-M-14_ was located on an IncFII2 plasmid. To further explore the genetic environment of the *bla*_CTX-M-14_ gene in isolate SP1, using the *bla*_CTX-M-14_ carrying contig as a query against the nr/nt database revealed sequence homology to the ~74 kb annotated *bla*_CTX-M-14_-positive IncFII2 plasmid pAC2901 (GenBank: KU987452) from *Citrobacter freundii* strain AC2901 (Fig. 2). Multiple sequence alignments demonstrated that DNA sequences between pSP1 and pAC2901 share >99% identity. Successful dissemination of *bla*_CTX-M-14_ among *Enterobacteriaceae* isolates from humans, animals and the environment has mainly been associated with IncK, IncF and IncI1 plasmids [38], and there has been only 1 report of a *bla*_CTX-M-14_ located on an IncFII2 plasmid in the English literature [39]. Here we report for the first time an IncFII2 *bl*a_CTX-M-14_-encoding plasmid in the genus *Shigella*.

### Pathogenesis and virulence factors

*Shigella flexneri* remains a public health concern throughout the world and its pathogenesis should be further investigated. We performed a BLASTP search against the VRDB database and found several known virulence factors. These virulence factors include *Shigella* extracellular protein A (*sepA*), glutamate decarboxylase (*gadA*), invasion plasmid antigen (*ipaH9.8*), long polar fimbriae (*lpfA*), hexosyltransferase homolog (*capU*), invasion protein *Shigella flexneri* (*ipaD*), serine protease autotransporters of *Enterobacteriaceae* (*pic*), VirF transcriptional activator (*virF*), and *Shigella* IgA-like protease homologue (*sigA*). The ability to withstand an acid-challenge of pH 2.5 by *S. flexneri* is a necessary virulence trait, which requires acid-induction of a functional GdaA in the stationary-growth phase [40]. IpaH9.8 is a member of the IpaH family of *Shigella*, which are encoded on the 220 kb virulence plasmid or chromosome and has been shown to be secreted into the host cell where it is targeted to the nucleus [41]. Moreover, LpfA has been linked to virulence in enterohemorrhagic *E. coli* [42]. Earlier reports indicated the SigA is cytopathic for HEp-2 cells and contributes to the intestinal fluid accumulation associated with *S. flexneri* infections [43]. Translocation of effector proteins into host cells and the surrounding space is a common strategy used by *S. flexneri* to target signaling pathways in the host cell [44], and IpaD and VirF are required to facilitate bacterial invasion of host cells [45]. More importantly, Pic is secreted by pathogenic Gram-negative bacteria through the autotransporter pathway and targets a broad range of human leukocyte adhesion proteins, which represent unique immune-modulating bacterial virulence factors [46]. These data suggest that isolate SP1 is potential pathogenic, which is consistent with the isolation of SP1 from a diarrhea patient.

### Comparative analysis with other *S. flexneri* strains

**Fig. 3:**
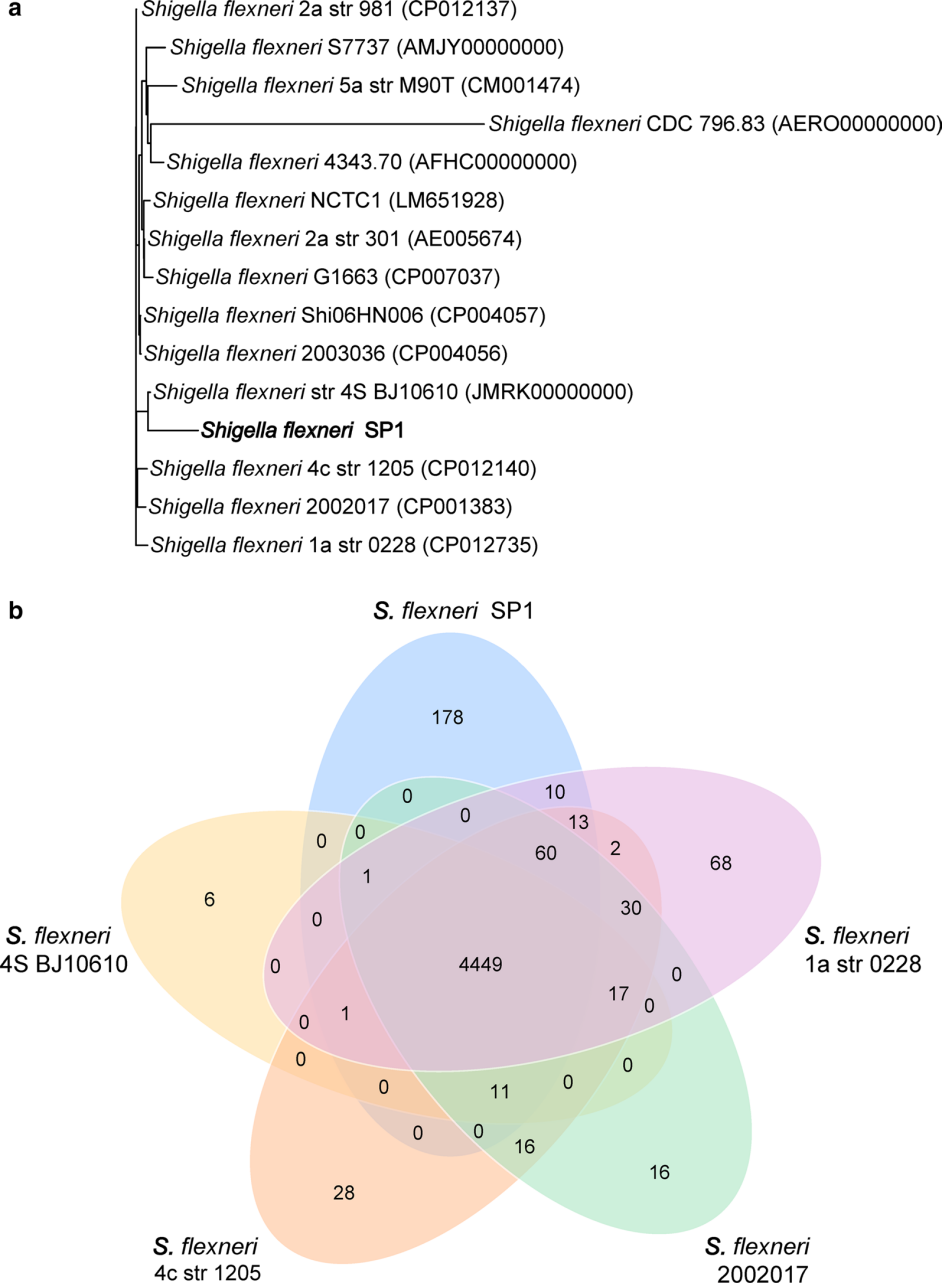
**a** Phylogenetic tree of *S. flexneri* strain SP1 with 14 other *S. flexneri* isolates. The tree was constructed based on based on alignments of orthologous genes. **b** Venn diagrams showing the orthologous groups in the five most closely related *S. flexneri* genomes. *Numbers* inside the Venn diagrams indicated the number of genes found to be shared among the indicated genomes

Based on genomes downloaded from the NCBI database, phylogenetic analysis was performed and the resulting tree topology was assessed to identify genetic relatedness between 14 *S. flexneri* isolates and strain SP1 (Fig. 3a). This revealed that SP1 is most closely related to *S. flexneri* str 4S BJ10610, which was also isolated from a severe diarrhea patient and was resistant to multiple drugs [47]. Other *S. flexneri* strains are also has highly similar, except for *S. flexneri* CDC 796.83, suggesting that *S. flexneri* strains show high similarity between different species. A previous study has highlighted that *S. flexneri* has a stable core genome that is equipped with a repertoire of virulence determinants that have enabled it to colonize, and persist, in multiple locations for hundreds of years [48]. A functional genomic comparison was performed between strain SP1 and its four most closely related neighbors: *S. flexneri* str 4S BJ10610 (JMRK00000000), *S. flexneri* 2002017 (CP001383), *S. flexneri* 4c str 1205 (CP012140), and *S. flexneri* 1a str 0228 (CP012735). The Venn diagram indicates the presence of 4449 core conserved genes present in the pan-genome of the analyzed *S. flexneri* isolates (Fig. 3b). Interestingly, a total of 178 strain-specific genes were identified in strain SP1.

## Conclusions

As more *S. flexneri* genomes have been sequenced in recent years, comparative genomic studies have progressed rapidly. So far, whole genome studies of *S. flexneri* have exclusively focused on the historical global spread and recent local persistence among these isolates. This work is the first description of the draft genome of a *bla*_CTX-M-14_-harbouring *S. flexneri* isolate and demonstrates the compares the genome of strain SP1 to other *S. flexneri* isolates. However, the data presented here is a preliminary report on the virulence profile and antibiotic resistance of *S. flexneri* strain SP1. Future studies involving more ESBL-encoding *S. flexneri* isolates from China are urgently needed to study the dynamics of the dissemination of ESBL genes, especially the complete sequence of plasmids carrying these ESBL genes.

## Additional files

**Additional file 1: Table S1.** Summary of *S. flexneri* strain SP1 genome.

**Additional file 2: Table S2.** Antimicrobial susceptibility profile of *S. flexneri* SP1 recovered from a patient with diarrhea.

**Additional file 3: Table S3.** Antibiotic resistance genes predicted in the *S. flexneri* strain SP1 genome.
