# Supplementary material for: Genome sequence of Shigella flexneri strain SP1, a diarrheal isolate that encodes an extended-spectrum β-lactamase (ESBL)
Source: Ann Clin Microbiol Antimicrob. 2017 May 12;16:37. doi: 10.1186/s12941-017-0212-2 (PMC5429569; doi:10.1186/s12941-017-0212-2)
Supplement: Supplementary file 1 — Additional file 1: Table S1. Summary of S. flexneri strain SP1 genome. [file 12941_2017_212_MOESM1_ESM.docx]

Table S1. Summary of *Shigella flexneri* strain SP1 genome

| Item | Value |
| --- | --- |
| Raw pair-end reads (kb) (*150bp) | 3,869,498 |
| Clean pair-end reads (kb) (*150bp) | 1,696,442 |
| Everage Coverage (X) | 110 |
| Total Length (bp) | 4,592,345 |
| Number of contigs | 345 |
| N50 Length (bp) | 33,394 |
| N90 Length (bp) | 6,377 |
| Max Length (bp) | 137,097 |
| Min Length (bp) | 507 |
| GC (%) | 50.46% |
