# Supplementary material for: Genome sequence of Shigella flexneri strain SP1, a diarrheal isolate that encodes an extended-spectrum β-lactamase (ESBL)
Source: Ann Clin Microbiol Antimicrob. 2017 May 12;16:37. doi: 10.1186/s12941-017-0212-2 (PMC5429569; doi:10.1186/s12941-017-0212-2)
Supplement: Supplementary file 2 — Additional file 2: Table S2. Antimicrobial susceptibility profile of S. flexneri SP1 recovered from a patient with diarrhea. [file 12941_2017_212_MOESM2_ESM.docx]

Table S2. Antimicrobial susceptibility profile of *Shigella flexneri* SP1 recovered from the patient with diarrhea

| Antimicrobial | MIC (μg/mL) | Interpretation |
| --- | --- | --- |
| Ampicillin | ≥32 | R |
| Amoxicillin/Clavulanic acid | 16 | R |
| Piperacillin/Tazobactam | ≤4 | S |
| Cefazolin | ≥64 | R |
| Cefoxitin | ≤4 | S |
| Ceftriaxone | ≥64 | R |
| Cefepime | 2 | S |
| Aztreonam | 2 | S |
| Ertapenem | ≤0.5 | S |
| Imipenem | ≤1 | S |
| Amikacin | 4 | S |
| Gentamicin | ≤1 | S |
| Tobramycin | ≤1 | S |
| Ciprofloxacin | 1 | S |
| Levofloxacin | 1 | S |
| Tigecycline | ≤0.5 | S |
| Nitrofurantoin | ≤16 | S |
| Trimethoprim | ≥320 | R |

R, resistant; S, susceptible. The MICs were interpreted according to the CLSI guidelines.
